# Comparative Evaluation of Machine Learning and Hyperparameter Optimization Methods for Low-Cost CO_2_ Sensor Calibration in Terms of Performance and Computational Cost

**DOI:** 10.3390/s26123671

**Published:** 2026-06-09

**Authors:** Eren Cihan Karsu Asal, Mehmet Taştan, Hayrettin Gökozan, Müge Erel-Özçevik, Yusuf Özçevik

**Affiliations:** 1 Department of Electric, Manisa Celal Bayar University, Manisa 45030, Turkey; hayrettin.gokozan@cbu.edu.tr; 2 Department of Electronics and Automation, Manisa Celal Bayar University, Manisa 45030, Turkey; mehmet.tastan@cbu.edu.tr; 3 Department of Software Engineering, Manisa Celal Bayar University, Manisa 45140, Turkey; muge.ozcevik@cbu.edu.tr (M.E.-Ö.); yusuf.ozcevik@cbu.edu.tr (Y.Ö.)

**Keywords:** low-cost CO_2_ sensors, machine learning, sensor calibration, hyperparameter optimization, Bayesian Optimization, grid search, random search

## Abstract

Low-cost sensors (LCSs) are increasingly used in air quality monitoring because of their affordability and scalability; however, their limited accuracy necessitates reliable calibration approaches. Although machine learning (ML)-based calibration methods have shown promising results, direct comparisons of hyperparameter optimization (HPO) strategies remain challenging due to differences in datasets, search spaces, and optimization budgets. In this study, ML models and HPO methods were evaluated within a standardized experimental framework developed on the AQ-MultiCal platform. Grid Search (GS), Random Search (RS), and Bayesian Optimization (BO) were implemented using identical hyperparameter search spaces and equal iteration budgets across both short-term and long-term real-world CO_2_ datasets obtained from five low-cost NDIR-based sensors. The results showed that tree-based models achieved strong baseline performance, whereas the k-nearest neighbors (kNN) model demonstrated the greatest improvement after optimization. The optimized kNN model reduced the average RMSE from 77.4 ppm to 54.4 ppm for the short-term dataset and from 37.3 ppm to 26.2 ppm for the long-term dataset. Although the HPO methods achieved comparable predictive accuracy, substantial differences were observed in computational cost. The proposed framework enables fair and reproducible comparison of HPO strategies while balancing predictive performance and computational efficiency in real-world sensor calibration applications.

## 1. Introduction

Air quality has become a critical issue for human health, environmental sustainability, and climate policy, particularly in urban environments. Increasing urbanization, traffic density, and industrial activities have intensified the need for air pollutant monitoring with high spatial and temporal resolution [1,2,3,4]. Although conventional reference measurement systems provide high measurement accuracy, their widespread deployment in large-scale and dense monitoring networks is limited by high installation and operational costs [5,6,7]. Consequently, LCSs have emerged as a scalable and cost-effective alternative for air quality monitoring applications in recent years [8].

However, LCS measurements are subject to substantial uncertainties arising from environmental conditions, sensor drift, inter-sensor variability, and non-linear response characteristics. In particular, environmental variables such as temperature and relative humidity can significantly influence sensor outputs, limiting the direct usability of raw sensor measurements and increasing the need for reliable calibration approaches [9]. Therefore, accurately modeling the complex relationships between reference measurements and LCS outputs has become a critical requirement for improving the reliability of LCS-based air quality monitoring systems. In recent years, ML-based calibration approaches have been widely adopted in sensor calibration studies due to their capability to model complex non-linear relationships [10,11,12]. Random Forest (RF), Gradient Boosting (GB), kNN, boosting-based ensemble learning models, and advanced feature extraction approaches have demonstrated promising performance across various low-cost gas sensing and calibration scenarios [13,14,15]. Nevertheless, considerable inconsistencies remain in the reported performances of different models throughout the literature. These variations indicate that model performance depends not only on the underlying algorithmic architecture but also on the characteristics of the dataset, data preprocessing procedures, validation strategies, and hyperparameter configurations. Because the performance of ML models is highly dependent on hyperparameter configurations, HPO has become a major research focus in ML-based sensor calibration studies [16,17]. Different optimization strategies, including GS, RS, and BO, have been widely employed in previous studies [18]. However, one of the primary methodological limitations in the existing literature is that HPO methods are often evaluated using different datasets, heterogeneous validation strategies, distinct hyperparameter search spaces, and unequal optimization budgets. In particular, variations in iteration numbers and search space structures used for the same model make direct and methodologically consistent comparisons between optimization strategies highly challenging, thereby substantially limiting cross-study comparability and reproducibility. Such methodological inconsistencies also hinder the transferability of calibration models to different sensor networks. Consequently, a standardized evaluation framework is needed to enable reliable validation of model performance across different datasets [19].

While a large proportion of existing studies primarily focus on predictive accuracy, the computational costs of optimization strategies and the trade-off between accuracy and computational efficiency are often insufficiently investigated. Furthermore, the influence of dataset size on model performance and optimization behavior has not been systematically examined in the literature. In particular, controlled comparative studies evaluating different optimization strategies using both short-term and long-term real-world field datasets remain very limited. In this study, the previously developed AQ-MultiCal platform [20] was extended to support the comparative evaluation of different HPO strategies under standardized experimental conditions. While the previous AQ-MultiCal study primarily focused on platform development and GS-based calibration analysis, the present study additionally incorporates RS and BO within the same experimental framework. All optimization methods were evaluated using identical hyperparameter search spaces and equal computational constraints. In addition to predictive performance, computational cost and optimization behavior were also comparatively analyzed using both short-term and long-term real-world datasets. In addition to conventional performance metrics, model behavior was further analyzed using residual distributions, prediction–reference agreement, and time-series tracking characteristics. This approach enabled a more comprehensive assessment of model behavior beyond aggregate accuracy measures.

The main contributions of this study can be summarized as follows:

- systematic comparison of different ML models and HPO strategies under standardized experimental conditions,
- evaluation of GS, RS, and BO methods using the same hyperparameter search space and identical iteration budgets,
- analysis of the impact of dataset size on model performance using both short-term and long-term real-world field datasets,
- assessment of computational cost in addition to predictive accuracy,
- development of a reproducible and integrated evaluation framework for sensor calibration applications.

To more clearly demonstrate the position of this study within the existing literature and highlight the primary contributions of the proposed approach, Table 1 presents a comparative overview of the methodological limitations in previous studies and the contributions of the proposed framework.

Table 1 summarizes the principal methodological limitations identified in the existing literature and demonstrates how the proposed AQ-MultiCal framework systematically and reproducibly addresses these challenges.

The remainder of this paper is organized as follows. Section 2 presents the datasets, data preprocessing procedures, modeling approach, and HPO process employed in this study. Section 3 comparatively analyzes the performances of different models and optimization strategies using both short-term and long-term datasets. In Section 4, the obtained findings are discussed within the context of the existing literature, and the main implications of the study are highlighted. Finally, Section 5 summarizes the study and outlines potential directions for future research.

## 2. Materials and Methods

In this study, a machine learning-based standardized evaluation framework was developed for the calibration of low-cost CO_2_ sensors. Data collection, data preprocessing, modeling, HPO, and performance evaluation procedures were conducted within the same experimental framework to enable the direct and consistent comparison of different models and optimization strategies.

### 2.1. Experimental Workflow

**Figure 1:**
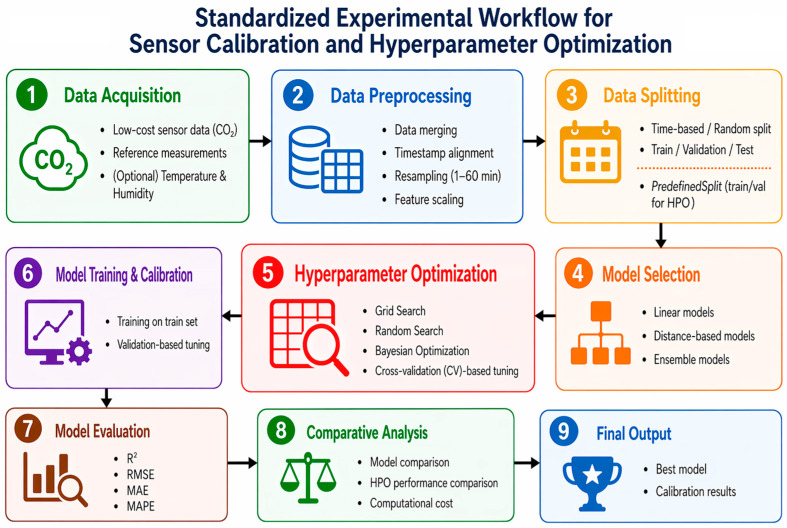
Standardized experimental workflow of the proposed sensor calibration framework, including data preprocessing, dataset partitioning, model training, hyperparameter optimization, and performance evaluation stages.

The overall workflow of the study is illustrated in Figure 1. The process consists of data collection, data preprocessing, data splitting, model training, HPO, and performance evaluation stages. All models and optimization methods were evaluated using the same data structure, data partitioning strategies, and performance metrics. This approach enables consistent comparison of different ML models and optimization strategies within the same experimental setting. During the data preprocessing stage, sensor and reference measurements were aligned based on timestamps, missing records were removed, and the datasets were prepared for subsequent analyses. The datasets were separately organized for both short-term and long-term analyses. This design allowed the systematic investigation of the influence of dataset size on model performance and optimization behavior.

During the HPO process, GS, RS, and BO strategies were employed. All optimization methods were evaluated using identical iteration budgets to ensure a controlled comparison of optimization behavior under equivalent computational constraints. Model performances were assessed using the coefficient of determination (R^2^) and root mean square error (RMSE) metrics. In addition, residual distributions, prediction–reference agreement, and time-series behaviors were also analyzed to provide a more comprehensive evaluation of model performance.

### 2.2. Data Collection

The dataset used in this study was obtained from five low-cost non-dispersive infrared (NDIR)-based CO_2_ sensors and a factory-calibrated commercial reference instrument (Dienmern DM72b, Dienmern (Shenzhen) Science & Technology Co., Ltd., Shenzhen, China) [20]. The reference instrument was factory-calibrated and operated according to the manufacturer-recommended conditions throughout the measurement campaign. All sensors were operated under the same environmental conditions throughout the data collection period, enabling controlled evaluation of inter-sensor performance variability.

The data collection campaign covered the period between January and February 2025. Measurements acquired at high temporal resolution were converted into one-minute averaged values. As a result, a total of 84,960 timestamp-aligned observations were obtained for each sensor together with the corresponding reference measurements.

To evaluate model performance under different data conditions, the dataset was examined using two separate configurations: short-term and long-term datasets. The long-term dataset covered the entire measurement period, whereas the short-term dataset was constructed as a subset extracted from the long-term dataset. This approach enabled a comparative analysis of the effects of dataset size on model learning behavior and generalization capability.

All analyses were conducted separately for each sensor, and performance metrics were calculated on a sensor-specific basis. The reported results correspond to the average performance across all sensors. This strategy reduced the influence of variability arising from individual sensor behavior on the overall findings. Detailed sensor-specific results are provided in the Supplementary Materials (Table S3).

**Figure 2:**
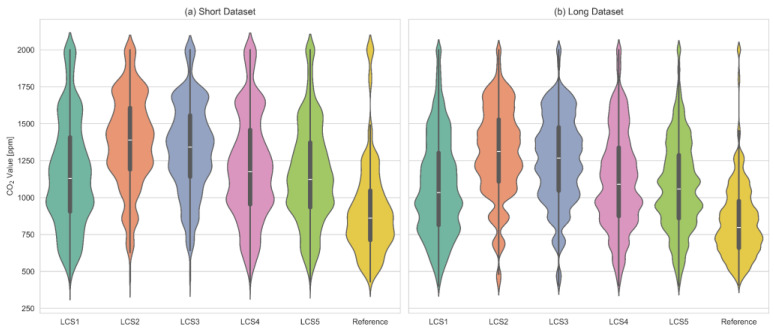
Distribution comparison between raw low-cost sensor measurements and reference CO_2_ concentrations for (**a**) short-term and (**b**) long-term datasets.

The distributions presented in Figure 2 indicate that the LCS measurements exhibit distributional characteristics that differ from those of the reference measurements. These discrepancies suggest that the direct use of raw sensor data may introduce various limitations and further highlight the necessity of ML-based calibration approaches.

### 2.3. Data Preprocessing, Data Splitting, and Input Variables

Prior to model development, the raw sensor data were processed using the preprocessing pipeline described in our previous study [20]. The preprocessing stage included temporal synchronization between low-cost sensor outputs and reference measurements, missing value handling, and signal-cleaning operations. Missing observations were treated using forward/backward filling, linear interpolation, and kNN-based imputation methods. The proportion of missing observations remained below 1% throughout the measurement campaign and therefore did not substantially affect the overall temporal continuity of the sensor signals. Timestamp alignment was additionally applied to ensure consistency between sensor and reference measurements. Reusing the same processed dataset also enabled direct comparison with the calibration results reported in the earlier study. The calibration models were constructed using raw CO_2_ measurements from the LCSs as predictor variables, while the corresponding reference instrument measurements were used as target outputs. Although the AQ-MultiCal framework supports the inclusion of auxiliary environmental variables such as temperature and relative humidity, these parameters were not incorporated into the present analyses. In our previous AQ-MultiCal study [20], the effects of environmental variables were systematically evaluated and were observed to produce model-dependent behavior under the evaluated experimental conditions. Since the primary objective of the present study was to establish a standardized and computationally balanced framework for comparative evaluation of HPO strategies, the analyses were conducted using only the raw CO_2_ sensor measurements as the primary predictor variable in order to ensure methodological consistency across all optimization experiments. Feature scaling was performed for algorithms sensitive to differences in variable magnitude. To avoid information leakage, scaling parameters were estimated exclusively from the training subset and subsequently applied to the validation and test data. No information from the validation or test partitions was used during model fitting or preprocessing parameter estimation. The short-term dataset consisted of measurements collected between 1 January and 7 January 2025, corresponding to 10,080 observations. Under limited data conditions, the dataset was partitioned into 80% training, 10% validation, and 10% test subsets in order to preserve sufficient data for model learning. The long-term dataset covered the January–February 2025 measurement campaign and contained 84,960 observations. For this dataset, a 70% training, 15% validation, and 15% test configuration was adopted to provide a more reliable assessment of generalization performance under larger data volumes. Hyperparameter optimization procedures were guided using the validation subset, whereas the test subset remained completely isolated until the final stage of performance assessment. All dataset partitions were generated using chronological (time-based) splitting, in which earlier observations were used for training, intermediate observations for validation, and later unseen observations for final testing. This strategy preserved the temporal structure of the sensor data and prevented future information leakage during model evaluation. To facilitate evaluation and ensure scientific transparency, the benchmark datasets used in this study are publicly available via the AQ-MultiCal GitHub repository.

### 2.4. Machine Learning Models

Various ML regression models were employed to evaluate the sensor calibration problem under different learning paradigms. The evaluated models included linear approaches (Ridge and ENet), the distance-based learning model kNN, tree-based models (DT (Decision Tree), RF, and GB), and boosting/ensemble learning-based models, including AdaB, XGB, LGBM, and CatB [14].

This diversity of models enabled the analysis of both linear and non-linear relationships between sensor measurements and reference observations under different learning strategies. Furthermore, evaluating different model categories within the same experimental framework allowed direct and consistent comparisons of model performance.

### 2.5. Hyperparameter Optimization

The performance of ML models is strongly influenced by hyperparameter configurations. Therefore, rather than relying on a single optimization strategy, GS, RS, and BO methods were comparatively evaluated in this study [32]. GS systematically explores all hyperparameter combinations within the predefined search space, whereas RS performs stochastic sampling from the hyperparameter space. In contrast, BO employs a probabilistic optimization strategy that guides the search process using information obtained from previous evaluations [24].

To ensure fair comparison among optimization strategies, all HPO methods were evaluated using the same hyperparameter search space and identical iteration budgets. The iteration budget was intentionally limited to 18 evaluations per model based on both computational feasibility and the structure of the predefined hyperparameter search spaces. The hyperparameter spaces were designed using the most influential parameters identified in previous analyses [20], and for most models, a compact 3 × 3 × 2 configuration corresponding to 18 candidate combinations was employed. This design enabled standardized and computationally balanced comparison among GS, RS, and BO methods while avoiding excessively large search spaces that could substantially increase computational cost without producing major accuracy gains. Consequently, the primary objective of this study was not exhaustive model-specific optimization, but fair and reproducible comparison of HPO strategies under equal computational constraints. The hyperparameter ranges used for each model are presented in Table 2.

The hyperparameter ranges were determined based on commonly used values reported in the literature, together with the principal parameters specific to each model architecture. The search spaces were designed to provide sufficient diversity to represent model learning behavior while avoiding excessively large search spaces that could introduce substantial computational overhead. All analyses were conducted within the same computational environment. In addition, the platform includes a user-configurable framework that allows custom hyperparameter selection and iteration budget specification.

R^2^ and RMSE were selected as the primary evaluation metrics to ensure consistent comparisons across different models and optimization strategies. Recent air quality studies emphasize that RMSE is one of the most reliable metrics for representing sensor calibration errors due to its sensitivity to large deviations and outlier behavior [33].

Modeling and optimization procedures were applied separately for each sensor to account for inter-sensor variability. Although all sensors operated under the same environmental conditions, differences in predictive performance were still observed among sensors. These findings suggest that sensor-specific calibration approaches may improve the reliability of LCS systems and provide more reliable overall performance estimates.

In addition to predictive accuracy, residual distributions, prediction–reference relationships, and time-series behaviors were also analyzed. Consequently, not only overall performance metrics but also error characteristics and temporal consistency of the models were evaluated.

An updated version of the platform, including enhanced data processing and HPO modules, was used in this study. All analyses were conducted using the Streamlit-based AQ-MultiCal platform implemented in the Python 3.12 environment. Scikit-learn, XGBoost, LightGBM, and CatBoost libraries were employed for the modeling processes, whereas BO procedures were implemented using the BayesSearchCV framework from the scikit-optimize library. Plotly was used for visualization tasks, while Pandas and NumPy libraries were utilized for data processing operations.

All analyses were performed on a workstation equipped with an Intel Core i7 processor and 16 GB RAM running the Windows 10 operating system. All models and optimization strategies were evaluated under the same experimental environment, and fixed random seed values were used during data partitioning procedures. Fixed random seeds were used to improve reproducibility of data partitioning and model comparison under the same computational setting.

## 3. Results

In this study, the performances of different ML models and HPO strategies were comparatively evaluated using short-term and long-term datasets. The analyses demonstrated that model architecture, dataset size, and optimization strategy have substantial effects on sensor calibration performance. The findings further indicate that not only predictive accuracy but also computational cost represents a critical criterion in the selection of models and optimization methods.

### 3.1. Comparison of Model Performance Using Default Parameters

Before applying HPO, the performances of different ML models using their default hyperparameter configurations were evaluated. The top three models with the lowest RMSE values for the short-term and long-term datasets are presented in Table 3.

The performance metrics presented in Table 3 reflect the mean values averaged across all five sensors, providing a generalized overview of the default model behaviors obtained from analyses conducted separately for each unit.

The results indicate that tree-based models maintain robust predictive performance even under default hyperparameter settings. Similar observations have been reported in previous sensor calibration studies, where ensemble-based approaches generally outperformed linear models under non-linear environmental conditions [19,32,34]. In addition, these models appeared more effective in compensating for fluctuations and noise commonly observed in LCS measurements.

For the short-term dataset, the lowest error was achieved by the RF model (RMSE = 68.2 ppm, R^2^ = 0.9169). CatB and LGBM models exhibited comparable performance levels. These findings suggest that tree-based and boosting-based models are capable of effectively capturing non-linear sensor behavior even under limited data conditions.

For the long-term dataset, RF again achieved the best overall performance (RMSE = 31.2 ppm, R^2^ = 0.9864). The DT model produced results very close to those of RF, whereas the kNN model demonstrated the third-best performance. Increasing dataset size improved the generalization performance of tree-based models, particularly RF and DT. Their strong predictive performance is likely related to their ability to model non-linear relationships while maintaining robustness against data variability.

When the short-term and long-term datasets are considered together, increasing dataset size appears to improve the performance of all models [35]. In particular, the reduction in the RF model RMSE from 68.2 ppm to 31.2 ppm indicates that larger datasets substantially enhance model generalization capability. Although the kNN model showed substantial improvement on the long-term dataset, it still produced higher error values than tree-based models under default hyperparameter settings. This finding indicates that the behavior of kNN is strongly influenced by hyperparameter selection.

Analysis times further revealed that computational cost generally increased with model complexity. Although the RF model achieved high predictive accuracy, it required substantially longer analysis times compared with several other models. In contrast, the LGBM model exhibited very low computational time. An appropriate balance between predictive accuracy and computational cost should be considered during model selection.

The effect of HPO on model performance was evaluated separately for the short-term and long-term datasets. The performance results of the optimized models are presented in Table 4. The findings indicate that the impact of HPO can vary substantially depending on model architecture, dataset size, and the selected optimization strategy.

The results presented in Table 4 indicate that the optimized kNN model achieved the greatest performance improvement for the short-term dataset. Under default hyperparameter settings, the kNN model produced an RMSE value of 77.4 ppm, which decreased to 54.4 ppm after HPO. A substantial improvement was similarly observed in the R^2^ metric. The marked RMSE reduction observed for kNN suggests that neighborhood size, distance metric, and weighting scheme played a decisive role in its calibration performance. Previous studies have emphasized that the performance of distance-based models such as kNN strongly depends on neighborhood structure and distance metrics [18]. In particular, optimization of the number of neighbors, distance metric, and weighting scheme may have enabled the model to better capture localized data density patterns.

Performance improvements were also observed for the RF and DT models following optimization. However, these improvements remained more limited compared with those achieved by the kNN model. The robust results obtained by RF under default hyperparameter settings suggest that the model already operated close to its optimal configuration for the underlying dataset structure. Furthermore, the relatively stable nature of ensemble-based learning approaches against data variability may have reduced the influence of hyperparameter changes on overall performance [36].

Only limited performance improvements were observed after HPO for certain boosting-based models, including GB, AdaBoost (AdaB), LGBM, and CatB. This behavior is likely related to the strong predictive capability of these models even under default hyperparameter settings [25]. In addition, the compact hyperparameter search spaces used in this study may have restricted exploration of parameter regions capable of producing larger behavioral variations. The impact of HPO varied considerably across different model architectures. Similar findings have also been reported in previous studies, where hyperparameter optimization did not consistently produce substantial accuracy improvements [34].

The results obtained from the long-term dataset suggest that increased data density enables models to more effectively learn temporal and environmental variations. Larger datasets may particularly improve the generalization capability of models based on localized pattern learning [32]. Although the RF model exhibited stronger performance under default hyperparameter settings, it remained slightly behind the optimized kNN model after HPO despite maintaining high predictive accuracy.

**Figure 3:**
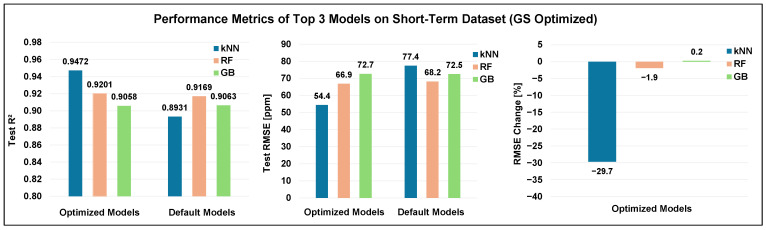
Comparative evaluation of optimized and default model performances for the short-term dataset in terms of R^2^, RMSE, and relative RMSE improvement.

Comparisons between optimized and default model performances for the short-term dataset are presented in Figure 3.

**Figure 4:**
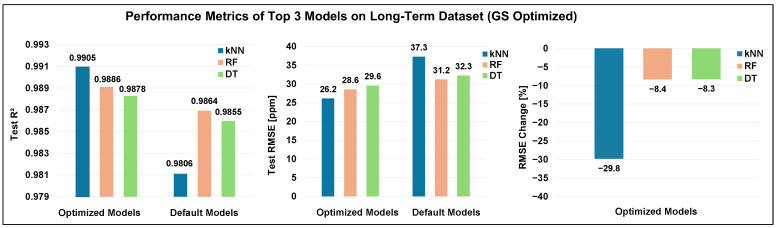
Comparative evaluation of optimized and default model performances for the long-term dataset in terms of R^2^, RMSE, and relative RMSE improvement.

Figure 3 presents the comparative performance changes between default and optimized model configurations for the short-term dataset. Comparisons between optimized and default model performances for the long-term dataset are presented in Figure 4.

Figure 4 illustrates the effect of dataset size on optimized model behavior. Furthermore, the absence of substantial accuracy differences among different HPO strategies may be associated with the ability of GS, RS, and BO methods to converge toward similar optimal regions within the low-dimensional and compact hyperparameter search spaces employed in this study.

Notably, under identical iteration budgets, the adaptive surrogate-based search advantage of BO appeared to remain limited. The convergence of different HPO strategies toward similar optimum regions under equal iteration constraints is likely related to the relatively small size of the hyperparameter search space. Similar studies in the literature have also reported that adaptive Bayesian approaches and stochastic sampling strategies can exhibit comparably competitive performance in budget-constrained AutoML processes [23]. Overall, the findings demonstrate that the impact of HPO varies depending on model architecture. While some models exhibited strong predictive performance even with default hyperparameter configurations, others achieved substantial improvements following optimization.

To further assess the effect of hyperparameter optimization, statistical significance analysis was performed for the best-performing model configuration. A one-sided Wilcoxon signed-rank test was conducted using the sensor-specific RMSE values obtained from the default and optimized kNN configurations. The results demonstrated a statistically significant reduction in prediction error following hyperparameter optimization (*p* = 0.03125).

In addition, repeated optimization runs produced consistent predictive performance metrics across all evaluated models because the experimental workflow was fully deterministic, with fixed dataset partitions, predefined validation structures, fixed random seed values, and identical hyperparameter search spaces. Minor variations were observed only in analysis duration, with average runtime differences across repeated runs remaining below 5% for all evaluated models. These variations can be attributed to system-level execution differences such as CPU scheduling and background computational load.

### 3.2. General Evaluation of Models and Optimization Strategies

**Figure 5:**
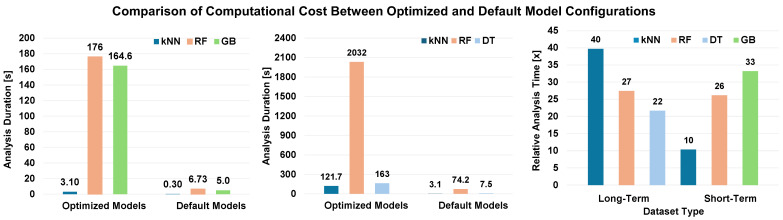
Comparison of computational costs between optimized and default model configurations for short-term and long-term datasets in terms of analysis duration and relative time increase.

The performance of the HPO strategies was evaluated in terms of both predictive accuracy and computational cost. Different optimization strategies produced broadly comparable predictive performances. The computational impact of HPO varied across model architectures. In particular, the differences in analysis time became increasingly pronounced as dataset size and model complexity increased. This observation highlights computational efficiency as an important criterion in the selection of optimization strategies. The obtained findings are further supported by the detailed performance comparisons presented in the Supplementary Materials (Tables S1 and S2). Figure 5 compares the analysis times (s) and relative time increases (x) of optimized and default model configurations for both short-term and long-term datasets.

The results presented in Figure 5 demonstrate that HPO substantially increased computational cost, particularly for tree-based models. For the long-term dataset, the analysis time of the RF model optimized using GS reached approximately 2032 s, whereas the default RF model required only 74.2 s. Similarly, the analysis time of the GS-optimized DT model increased to 163 s, while the default DT model required only 7.5 s. These findings indicate that the HPO process can introduce considerable computational overhead, especially for models involving large numbers of decision trees. Increasing dataset size significantly increased computational cost due to repeated model training during the optimization process. In contrast, the relative increase in analysis time remained more moderate for the kNN model. For the long-term dataset, the GS-optimized kNN model required 121.7 s of analysis time, compared with 3.1 s for the default configuration. Despite the additional computational cost, the optimized kNN model achieved the greatest improvement in predictive performance, providing a more favorable balance between accuracy and computational efficiency.

Examination of the relative time increases further revealed that the impact of HPO varied depending on model architecture. For the long-term dataset, the computational time increased by approximately 40-fold for the kNN model, 27-fold for the RF model, and 22-fold for the DT model. For the short-term dataset, the highest relative increase was observed for the GB model, with an approximately 33-fold increase in analysis time. Similarly, relative increases of approximately 26-fold, 22-fold, and 10-fold were observed for the RF, DT, and kNN models, respectively. Both dataset size and model complexity played important roles in determining the computational cost of HPO.

GS provided stable optimization behavior through systematic exploration of the hyperparameter space, whereas RS and BO achieved comparable predictive performance using more flexible search strategies. In several cases, RS reached competitive accuracy levels with lower computational cost, suggesting that stochastic sampling approaches can provide efficient exploration within constrained search spaces. Overall, computational efficiency emerged as a major differentiating factor among optimization strategies. These findings highlight the importance of balancing predictive performance and computational efficiency during optimization strategy selection. Although the present study focused on optimization-related computational cost, deployment-oriented metrics such as inference latency, memory consumption, and model size may also be important for real-time or resource-constrained sensor calibration applications.

**Figure 6:**
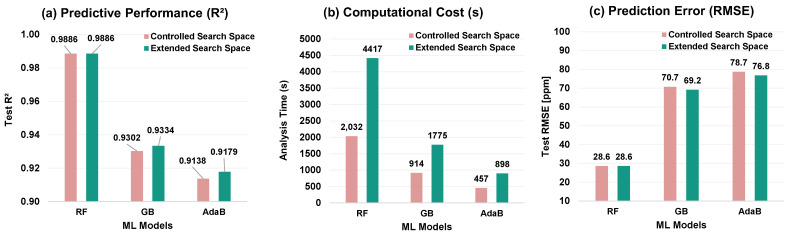
Comparison of controlled and extended hyperparameter search spaces for RF, GB, and AdaBoost models in terms of (**a**) predictive performance (R^2^), (**b**) computational cost, and (**c**) RMSE.

Figure 6 compares the effects of controlled and extended hyperparameter search spaces on the RF, GB, and AdaBoost models in terms of (a) predictive performance (R^2^), (b) computational cost, and (c) prediction error (RMSE). The fixed optimization budget used in this study was determined based on the extended hyperparameter search space analyses conducted in the previous study [20]. In the previous study, the RF, GB, and AdaBoost models, which exhibited the highest computational costs among the ensemble-based models, were evaluated using 60, 48, and 36 iterations, respectively. As illustrated in Figure 6b, expanding the hyperparameter search space and increasing the iteration count substantially increased the computational cost. However, as shown in Figure 6a,c, the improvements obtained in the performance metrics remained relatively limited. For instance, although the analysis time of the RF model increased by approximately 117%, no meaningful improvement was observed in the R^2^ value, while the RMSE improvement remained at approximately 0.02%. Similarly, for the GB model, the computational cost increased by approximately 94%, whereas the R^2^ value improved by only about 0.33% and the RMSE decreased by approximately 2.2%. In the AdaBoost model, despite an approximately 96% increase in analysis time, the improvement in R^2^ remained limited to approximately 0.45%, while the RMSE improved by about 2.4%. These findings demonstrate that higher optimization budgets do not always provide meaningful performance gains and that the trade-off between computational cost and predictive improvement should be carefully considered during hyperparameter optimization. Furthermore, long optimization times may represent a significant disadvantage in computationally constrained applications such as online calibration, real-time air quality monitoring systems, and embedded platforms with limited hardware capacity. Accordingly, a common 18-iteration optimization budget was adopted for all models to ensure direct and methodologically consistent comparison among different HPO strategies. Although adaptive stopping criteria may further reduce computational overhead for computationally expensive models such as RF, GB, and AdaBoost, fixed optimization budgets were intentionally maintained in this study.

### 3.3. Graph-Based Model Performance Analysis

**Figure 7:**
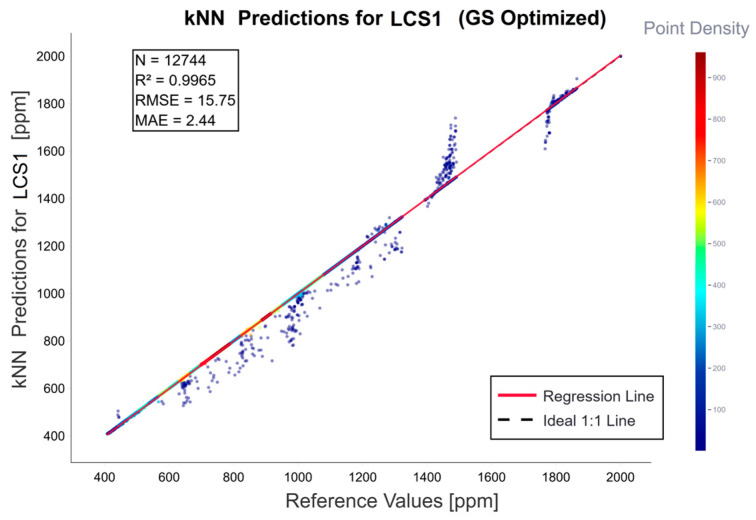
Prediction–reference agreement for the GS-optimized kNN model using the long-term dataset of LCS1.

The best overall performance after HPO was achieved by the kNN model optimized using GS. Therefore, graph-based analyses were conducted on the long-term dataset to enable a more detailed evaluation of model behavior. In this section, focusing on LCS1 as a representative unit allows for a clearer visualization of the model’s calibration potential. As illustrated in Figure 7, the sample size (N = 12,744) represents the isolated 15% test partition of the total long-term observations for this specific sensor unit. While the study generally presents average performance metrics across all sensors to reduce the influence of individual variability, providing a detailed assessment at the single-sensor level facilitates an in-depth understanding of error characteristics and temporal tracking reliability. Detailed sensor-specific results for the remaining units are provided in the Supplementary Materials (Table S3).

As illustrated in Figure 7, the predicted CO_2_ values for LCS1 using the long-term dataset are tightly distributed around the ideal 1:1 line. The strong agreement between the regression and ideal lines, reflected by an R^2^ of 0.9965, indicates strong consistency between predicted and reference values under the GS-optimized kNN model. Although a marginal increase in dispersion can be observed at elevated CO_2_ concentrations, the overall prediction–reference agreement remains robust across the entire measurement range. These results suggest that the GS-optimized approach can effectively compensate for sensor response variability over extended monitoring periods.

**Figure 8:**
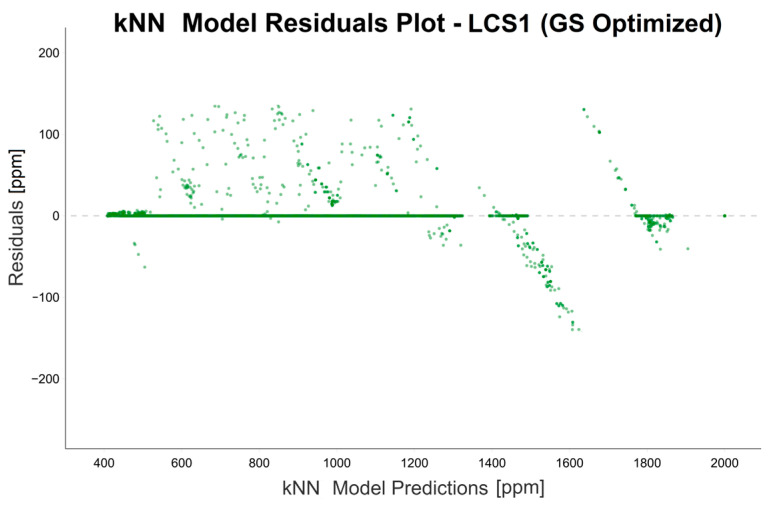
Residual distribution of the GS-optimized kNN model for the LCS1 long-term dataset.

The residual distribution of the GS-optimized kNN model for LCS1 is presented in Figure 8. The residuals are centered around the zero line across the long-term monitoring period, illustrating the error characteristics of the model under various CO_2_ concentrations. While the dispersion remains consistent across the majority of the measurement range, the residual distribution provides an overall indication of the model’s behavior relative to the sensor response. This visual representation allows for an assessment of the calibration alignment throughout the entire dataset without the influence of inter-sensor variability.

**Figure 9:**
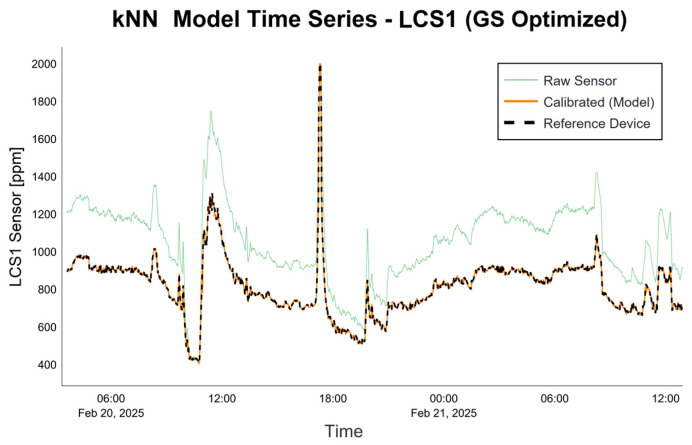
Time-series comparison of raw sensor measurements, calibrated outputs, and reference CO_2_ concentrations for LCS1 using the GS-optimized kNN model.

The temporal tracking capability of the GS-optimized kNN model for LCS1 is illustrated in Figure 9. The time-series plot demonstrates that the calibrated model predictions successfully follow the overall temporal trends observed in the reference measurements. In particular, the ability of the model outputs to exhibit dynamic behavior identical to the reference signal—especially during the abrupt variation regions and peak events—indicates that the machine learning-based calibration approach can effectively capture complex temporal patterns.

Despite the substantial correction of the raw sensor signal, limited smoothing effects can be observed in certain high-peak regions where model predictions partially attenuate rapid concentration fluctuations. Nevertheless, the optimized kNN model successfully preserved the overall temporal dynamics and maintained substantially closer agreement with the reference measurements throughout the majority of the monitoring period.

## 4. Discussion

The findings demonstrate that the effects of hyperparameter optimization vary considerably across machine learning architectures. Under default parameter settings, tree-based models such as RF and DT demonstrated stable and robust predictive performance across both datasets. However, the changes observed after hyperparameter optimization suggest that the sensitivity of model performance to parameter tuning varies considerably across different learning architectures.

The substantial performance improvement observed for the kNN model indicates that its calibration capability is highly sensitive to hyperparameter selection. Optimization of neighborhood size, distance metric, and weighting strategy appeared to significantly improve the model’s ability to represent localized non-linear patterns within the sensor data. After optimization, the kNN model achieved the lowest RMSE values across both datasets, demonstrating that neighborhood-based learning is highly responsive to hyperparameter tuning in low-cost sensor calibration. Similar findings have also been reported in previous sensor calibration studies involving distance-based learning approaches [37].

In contrast, the strong performance of RF and DT models even under default hyperparameter settings suggests that these models may exhibit greater robustness to dataset characteristics and lower sensitivity to hyperparameter variations [38].

From the perspective of optimization strategies, GS, RS, and BO produced largely comparable predictive performances under the constrained search spaces employed in this study. Similar observations have been reported in previous studies, where the performance differences among optimization strategies remained limited in relatively low-dimensional search problems [25]. In contrast, the advantages of BO are generally more pronounced in higher-dimensional optimization tasks involving broader search spaces and more complex parameter interactions. Under the experimental conditions considered in this study, model architecture appeared to play a more prominent role in performance variation than the optimization strategy itself.

Recent studies have also demonstrated the potential of advanced deep learning-based approaches for gas prediction and sensor calibration applications, including temporal convolutional networks, stacking-based neural architectures, and lightweight gated aggregation models [39,40,41]. Such approaches may provide strong predictive capability for complex temporal sensor data and high-dimensional feature extraction tasks. However, these models generally require substantially larger datasets, increased computational resources, and more complex training and optimization procedures compared with conventional ML-based approaches. In contrast, the framework evaluated in the present study was designed to provide computationally controlled, reproducible, and practically applicable comparisons among conventional ML-based calibration strategies under standardized experimental conditions.

This behavior is likely related to the compact and low-dimensional search spaces employed in this study. Nevertheless, substantial differences were observed among the methods in terms of computational cost. Analysis times increased considerably with growing dataset size and model complexity. While the HPO process introduced substantial computational overhead for models such as RF and GB, the RS strategy was, in certain cases, able to achieve comparable accuracy levels with lower analysis times. These results suggest that computational efficiency should also be considered during model selection for practical sensor calibration applications. Larger datasets improved model generalization performance, particularly for the kNN architecture, suggesting that data density plays an important role in localized pattern learning.

Although residuals were mostly centered around zero, increased variance at high CO_2_ concentrations suggests that modeling extreme concentration regions remains challenging. Furthermore, the time-series analyses revealed that the optimized kNN model was capable of successfully tracking the overall temporal trends of the reference signal. In this study, conducting analyses separately for each sensor and reporting average performance values contributed to a more representative evaluation of model behavior. This approach reduced the influence of inter-sensor measurement variability on the overall findings.

Despite the promising results obtained in this study, several limitations should be acknowledged. Although the long-term dataset enabled evaluation under longer-term monitoring conditions, additional multi-season deployments may provide further insight into long-term environmental variability and sensor drift behavior. The present study was conducted using a single NDIR-based sensor family under controlled environmental conditions in order to enable standardized comparison among different ML models and HPO strategies. Although the obtained findings demonstrate strong comparative performance within this experimental framework, future studies may further evaluate the proposed approach across different sensing technologies, climatic regions, and independent external datasets.

In addition, although controlled comparisons among HPO strategies were enabled using fixed iteration budgets and common search spaces, the relatively compact optimization settings may have limited the exploration capability of adaptive approaches such as Bayesian Optimization. Statistical significance analyses between optimization strategies were also not included in the present study. Future studies may further improve the robustness of ML-based calibration frameworks by incorporating larger multi-site datasets, broader search spaces, additional air pollutants such as PM2.5, and more comprehensive statistical evaluation methods.

## 5. Conclusions

In this study, different ML models and HPO strategies were systematically evaluated for the calibration of low-cost CO_2_ sensors using both short-term and long-term datasets. The results showed that model performance was influenced not only by the selected algorithm but also by dataset characteristics and hyperparameter configurations. While tree-based models such as RF and DT demonstrated strong baseline performance, the kNN model achieved the greatest improvement after optimization.

The comparison of HPO strategies revealed that GS, RS, and BO generally achieved comparable predictive accuracy under the evaluated conditions. However, substantial differences were observed in computational cost, particularly for computationally intensive ensemble-based models, highlighting the need to balance accuracy and execution time in resource-limited applications. In addition, the long-term dataset contributed to improved model stability, while the graph-based analyses demonstrated that the optimized kNN model could effectively track temporal variations in the reference measurements.

Several limitations of this study should also be acknowledged. The analyses were limited to a specific sensor family and environmental conditions, which may restrict the generalizability of the findings to different monitoring scenarios. In addition, the relatively compact hyperparameter search spaces and fixed iteration budgets may have limited the exploration capability of adaptive optimization approaches such as Bayesian Optimization. External validation using independent datasets collected under different environmental conditions was also not performed. Future studies may further improve the robustness and transferability of ML-based calibration models by incorporating larger multi-site datasets, broader search spaces, and additional statistical evaluation methods. Overall, the study highlights the importance of jointly considering predictive performance, computational efficiency, and dataset characteristics in ML-based sensor calibration. In future real-time calibration applications, deployment-oriented factors such as inference speed, memory usage, and model complexity may also play an important role in model selection.

## Figures and Tables

**Table 1:** Comparison of Existing Literature Limitations and the Contributions of the Proposed AQ-MultiCal Framework.

Research Focus	Limitations in the Literature	Contributions of the Proposed Framework	Relevant Literature
Lack of standardized ML evaluation	Different datasets, data preprocessing procedures, and hyperparameter search spaces reduce cross-study comparability.	A standardized experimental framework was established using a common dataset, fixed data-splitting ratios, and a shared hyperparameter search space.	[21,22]
Lack of fair comparison among HPO methods	HPO methods are commonly evaluated using different iteration numbers and optimization budgets.	GS, RS, and BO methods were compared using identical iteration budgets and the same hyperparameter search space.	[23,24,25,26,27]
Limited evaluation of computational cost	Most studies primarily focus on predictive performance.	Computational cost was evaluated alongside model accuracy.	[28,29,30]
Limited investigation of dataset size effects	The effects of short-term and long-term datasets are not systematically compared.	Both short-term and long-term datasets were evaluated within the same framework.	[31,32]
Fragmented calibration workflows	Data preprocessing, modeling, and optimization processes are conducted using separate tools and environments.	An integrated analytical environment was provided through the AQ-MultiCal platform.	This Study

**Table 2:** Hyperparameter configurations and iteration numbers used for the ML models.

Model Name	Hyperparameters (Values)	Iter.	Model Name	Hyperparameters (Values)	Iter.
RF	n_estimators:[100,200,300]; max_depth:[10,30,None]; min_samples_split:[2,5]	18	DT	max_depth:[5,10,None]; min_samples_split:[2,5,10]; min_samples_leaf:[1,2]	18
GB	n_estimators:[100,200,300]; learning_rate:[0.05,0.1,0.2]; max_depth:[3,5]	18	AdaB	n_estimators:[50,100,200]; learning_rate:[0.05,0.1,0.2]; loss:[‘linear’,’square’]	18
kNN	n_neighbors:[20,30,40]; weights:[‘uniform’,’distance’]; metric:[‘euclidean’,’manhattan’,’chebyshev’]	18	XGB	n_estimators:[100,200,300]; learning_rate:[0.05,0.1,0.2]; max_depth:[3,5]	18
EN	alpha:[0.01,0.1,1.0]; l1_ratio:[0.1,0.5,0.9]; fit_intercept:[True,False]	18	LGBM	n_estimators:[100,200,300]; learning_rate:[0.05,0.1,0.2]; num_leaves:[20,40]	18
Ridge	alpha:[0.01,0.1,1.0]; fit_intercept:[True,False]; solver:[‘auto’,’svd’,’lsqr’]	18	CB	n_estimators:[100,200,300]; learning_rate:[0.05,0.1,0.2]; depth:[4,10]	18

**Table 3:** Performance of the top three ML models with the lowest RMSE values using default hyperparameters for the short-term and long-term datasets.

Data Set	Rank	Model	Analysis Time (s)	Test R^2^	Test RMSE (ppm)
short-term	1	RF	6.7	0.9169	68.2
	2	CatB	9.6	0.9073	72.1
	3	LGBM	0.6	0.9067	72.3
long-term	1	RF	74.2	0.9864	31.2
	2	DT	7.5	0.9855	32.3
	3	kNN	3.1	0.9806	37.3

**Table 4:** Performance of the top three ML models with the lowest RMSE values after HPO for the short-term and long-term datasets.

Data Set	Rank	Model	Opt. Method	Analysis Time (s)	Test R^2^	Test RMSE (ppm)
short-term	1	kNN	GS	3.1	0.9472	54.4
	2	RF	GS	176	0.9201	66.9
	3	GB	GS	165	0.9058	72.7
long-term	1	kNN	RS	42.7	0.9905	26.2
	2	RF	BO	1798	0.9886	28.6
	3	DT	RS	39.1	0.9879	29.4

## Data Availability

The source code of the AQ-MultiCal platform developed in this study is publicly available on GitHub at https://github.com/tastan45/AQ-MultiCal_v1, accessed on 17 May 2026. The interactive web-based application can also be accessed at https://aq-multicalv1-mg6hyos8zzxx54rwhd8qwt.streamlit.app/, accessed on 17 May 2026.

## Supplementary Materials

The following supporting information can be downloaded at: https://www.mdpi.com/article/10.3390/s26123671/s1, Table S1: Comparison of different HPO strategies in terms of model prediction accuracy and computational efficiency on the long-term CO_2_ dataset; Table S2: Comparison of different HPO strategies in terms of model prediction accuracy and computational efficiency on the short-term CO_2_ dataset, Table S3: Sensor-wise evaluation of the kNN model performance on the long-term CO_2_ dataset under different hyperparameter optimization strategies.
