# Supplementary material for: Comparative Evaluation of Machine Learning and Hyperparameter Optimization Methods for Low-Cost CO2 Sensor Calibration in Terms of Performance and Computational Cost
Source: Sensors (Basel). 2026 Jun 9;26(12):3671. doi: 10.3390/s26123671 (PMC13307083; doi:10.3390/s26123671)
Supplement: Supplementary file 1 [file sensors-26-03671-s001.zip › sensors-4353515-supplementary.pdf]

Table S1. Comparison of different HPO strategies in terms of model prediction accuracy and computational efficiency on the long-term CO_2 dataset.

Model Name	Opt. Method	Analysis Duration (s)	Test R^2	% Change R^2	RMSE	% Change RMSE	Optimization HyperParameters Value
kNN	**-**	3.1	0.9806		37.31		**dash**
	GS	121.7	0.9905	1.00	26.17	-29.8	metric: chebyshev, n_neighbors: 40, weights: distance
	RS	42.8	0.9905	1.00	26.17	-29.8	metric: manhattan, n_neighbors: 40, weights: distance
	BO	60.9	0.9905	1.00	26.17	-29.8	metric: manhattan, n_neighbors: 40, weights: distance
RF	-	74.2	0.9864		31.22		dash
	GS	2032.1	0.9886	0.22	28.60	-8.38	max_depth: None, min_samples_split: 2, n_estimators: 300
	RS	1779.4	0.9885	0.21	28.73	-7.97	max_depth: None, min_samples_split: 4, n_estimators: 234
	BO	1798.3	0.9886	0.22	28.60	-8.38	max_depth: None, min_samples_split: 2, n_estimators: 300
GB	-	29.7	0.9263		72.70		dash
	GS	914.0	0.9302	0.42	70.75	-2.69	learning_rate: 0.2, max_depth: 5, n_estimators: 300
	RS	476.2	0.9271	0.09	72.30	-0.55	learning_rate: 0.1848, max_depth: 3, n_estimators: 239
	BO	697.5	0.9287	0.26	71.52	-1.62	learning_rate: 0.2, max_depth: 4, n_estimators: 300
LGBM	-	18.9	0.9254		73.19		dash
	GS	49.3	0.9256	0.03	73.05	-0.19	learning_rate: 0.1, n_estimators: 100, num_leaves: 31
	RS	53.2	0.9256	0.03	73.05	-0.19	learning_rate: 0.0734, n_estimators: 149, num_leaves: 27
	BO	50.4	0.9256	0.03	73.05	-0.19	learning_rate: 0.0833, n_estimators: 200, num_leaves: 20
CatB	-	72.6	0.9252		73.27		-
	GS	60.2	0.9250	-0.02	73.36	0.13	depth: 6, learning_rate: 0.1, n_estimators: 200
	RS	49.3	0.9250	-0.02	73.36	0.13	depth: 4, learning_rate: 0.0734, n_estimators: 149
	BO	58.8	0.9250	-0.02	73.36	0.13	depth: 6, learning_rate: 0.1, n_estimators: 200
XGB	-	1.7	0.9203		75.60		-
	GS	31.0	0.9213	0.10	75.17	-0.58	learning_rate: 0.1, max_depth: 7, n_estimators: 200
	RS	24.2	0.9213	0.10	75.16	-0.58	learning_rate: 0.0724, max_depth: 7, n_estimators: 122
	BO	44.7	0.9213	0.10	75.15	-0.60	learning_rate: 0.0657, max_depth: 5, n_estimators: 200
DT	-	7.5	0.9855		32.29		-
	GS	162.6	0.9878	0.23	29.60	-8.31	max_depth: None, min_samples_leaf: 1, min_samples_split: 5
	RS	39.1	0.9879	0.25	29.45	-8.79	max_depth: None, min_samples_leaf: 1, min_samples_split: 4
	BO	59.8	0.9878	0.23	29.63	-8.22	max_depth: None, min_samples_leaf: 1, min_samples_split: 2
AdaB	-	15.1	0.9183		76.59		-
	GS	457.3	0.9138	-0.49	78.67	2.71	learning_rate: 0.2, loss: square, n_estimators: 50
	RS	301.6	0.9139	-0.48	78.62	2.66	learning_rate: 0.0217, loss: square, n_estimators: 183
	BO	314.8	0.9138	-0.49	78.67	2.71	learning_rate: 0.2, loss: square, n_estimators: 50
Ridge	-	0.5	0.8356		108.62		-
	GS	5.7	0.8362	0.08	108.40	-0.20	alpha: 0.01, fit_intercept: True, solver: auto
	RS	1.6	0.8362	0.08	108.40	-0.20	alpha: 0.0106, fit_intercept: True, solver: auto
	BO	7.3	0.8362	0.08	108.40	-0.20	alpha: 0.01, fit_intercept: True, solver: auto
ENet	-	0.5	0.7250		140.48		-
	GS	2.9	0.8362	15.34	108.42	-22.8	alpha: 0.01, fit_intercept: True, l1_ratio: 0.9
	RS	1.8	0.8361	15.34	108.43	-22.8	alpha: 0.0108, fit_intercept: True, l1_ratio: 0.8725
	BO	7.4	0.8362	15.34	108.42	-22.8	alpha: 0.01, fit_intercept: True, l1_ratio: 0.9

Table S2. Comparison of different HPO strategies in terms of model prediction accuracy and computational efficiency on the short-term CO_2 dataset.

Model Name	Opt. Method	Analysis Duration (s)	Test R^2	% Change (R^2)	RMSE [ppm]	% Change (RMSE)	Optimization HyperParameters Value
kNN	-	0.30	0.8931		77.4		-
	GS	3.1	0.9472	6.1	54.4	-29.7	metric: euclidean, n_neighbors: 40, weights: distance
	RS	4.0	0.9472	6.1	54.4	-29.7	metric: chebyshev, n_neighbors: 40, weights: distance
	BO	12.5	0.9472	6.1	54.4	-29.7	metric: chebyshev, n_neighbors: 40, weights: distance
RF	-	6.73	0.9169		68.2		-
	GS	176	0.9201	0.4	66.9	-1.9	max_depth: 30, min_samples_split: 2, n_estimators: 200
	RS	160	0.9201	0.4	66.9	-1.9	max_depth: 30, min_samples_split: 2, n_estimators: 188
	BO	171	0.9201	0.3	66.9	-1.9	max_depth: 30, min_samples_split: 2, n_estimators: 216
GB	-	4.96	0.9063		72.5		-
	GS	165	0.9058	-0.1	72.7	0.2	learning_rate: 0.2, max_depth: 5, n_estimators: 300
	RS	87.1	0.9048	-0.2	73.0	0.7	learning_rate: 0.1314, max_depth: 5, n_estimators: 120
	BO	249	0.9053	-0.1	72.8	0.5	learning_rate: 0.1500, max_depth: 5, n_estimators: 300
LGBM	-	0.58	0.9067		72.3		-
	GS	6.89	0.9045	-0.2	73.1	1.2	learning_rate: 0.05, n_estimators: 100, num_leaves: 20
	RS	6.84	0.9047	-0.2	73.1	1.1	learning_rate: 0.0933, n_estimators: 87, num_leaves: 21
	BO	18.9	0.9046	-0.2	73.1	1.1	learning_rate: 0.0278, n_estimators: 188, num_leaves: 22
CatB	-	9.61	0.9073		72.1		-
	GS	12.2	0.9042	-0.3	73.3	1.6	depth: 6, learning_rate: 0.1, n_estimators: 200
	RS	14.5	0.9044	-0.3	73.2	1.5	depth: 6, learning_rate: 0.0962, n_estimators: 180
	BO	31.5	0.9045	-0.3	73.2	1.5	depth: 4, learning_rate: 0.0221, n_estimators: 200
XGB	-	0.38	0.9048		73.0		-
	GS	4.25	0.9029	-0.2	73.7	1.0	learning_rate: 0.05, max_depth: 5, n_estimators: 100
	RS	3.87	0.9030	-0.2	73.7	1.0	learning_rate: 0.0285, max_depth: 5, n_estimators: 157
	BO	17.5	0.9031	-0.2	73.7	0.9	learning_rate: 0.0257, max_depth: 6, n_estimators: 190
DT	-	0.36	0.9047		73.1		-
	GS	1.21	0.9012	-0.4	74.4	1.8	max_depth: 5, min_samples_leaf: 1, min_samples_split: 2
	RS	1.56	0.9012	-0.4	74.4	1.8	max_depth: 5, min_samples_leaf: 1, min_samples_split: 8
	BO	9.30	0.9012	-0.4	74.4	1.8	max_depth: 5, min_samples_leaf: 2, min_samples_split: 8
AdaB	-	1.25	0.9015		74.3		-
	GS	82.9	0.8925	-1.0	77.6	4.4	learning_rate: 0.05, loss: exponential, n_estimators: 100
	RS	48.9	0.8992	-0.3	75.1	1.1	learning_rate: 0.1716, loss: exponential, n_estimators: 63
	BO	113.1	0.8949	-0.7	76.7	3.3	learning_rate: 0.0901, loss: exponential, n_estimators: 75
Ridge	-	0.06	0.8050		104.5		-
	GS	0.29	0.8122	0.9	102.6	-1.8	alpha: 100.0, fit_intercept: True, solver: svd
	RS	1.00	0.8116	0.8	102.8	-1.7	alpha: 17.12, fit_intercept: True, solver: svd
	BO	7.1	0.8122	0.9	102.6	-1.8	alpha: 100.0, fit_intercept: True, solver: auto
ENet	-	0.07	0.7700		113.5		-
	GS	0.36	0.8308	7.9	97.4	-14.2	alpha: 1.0, fit_intercept: True, l1_ratio: 0.9
	RS	0.36	0.8229	6.9	99.6	-12.2	alpha: 0.4362, fit_intercept: True, l1_ratio: 0.4603
	BO	8.7	0.8286	7.6	98.0	-13.7	alpha: 0.7712, fit_intercept: True, l1_ratio: 0.7576

Table S3. Sensor-wise evaluation of the kNN model performance on the long-term CO_2 dataset under different hyperparameter optimization strategies.

Sensor	Test MAE [ppm]	Test MAPE [%]	Test R^2	Test RMSE [ppm]	Optimization Method
LCS1	8.5	0.78	0.9889	28.2	-
LCS2	12.4	1.40	0.9842	33.7	
LCS3	14.8	1.53	0.9781	39.7	
LCS4	9.5	0.85	0.9863	31.4	
LCS5	16.1	1.30	0.9655	49.7	
LCS1	2.4	0.23	0.9965	15.8	GridSearch
LCS2	3.2	0.33	0.9946	19.6	
LCS3	4.1	0.36	0.9918	24.2	
LCS4	3.6	0.28	0.9886	28.7	
LCS5	6.0	0.43	0.9807	37.2	
LCS1	2.4	0.23	0.9965	15.8	Random Search
LCS2	3.2	0.33	0.9946	19.6	
LCS3	4.1	0.36	0.9918	24.2	
LCS4	3.6	0.28	0.9886	28.7	
LCS5	6.0	0.43	0.9807	37.2	
LCS1	2.4	0.23	0.9965	15.8	Bayesian
LCS2	3.2	0.33	0.9946	19.6	
LCS3	4.1	0.36	0.9918	24.2	
LCS4	3.6	0.28	0.9886	28.7	
LCS5	6.0	0.43	0.9807	37.2	
